# Transcription Factors Expressed in Mouse Cochlear Inner and Outer Hair Cells

**DOI:** 10.1371/journal.pone.0151291

**Published:** 2016-03-14

**Authors:** Yi Li, Huizhan Liu, Cody L. Barta, Paul D. Judge, Lidong Zhao, Weiping J. Zhang, Shusheng Gong, Kirk W. Beisel, David Z. Z. He

**Affiliations:** 1 Department of Otorhinolaryngology—Head and Neck Surgery, Beijing Tongren Hospital, Capital Medical University, Beijing, 100730, China; 2 Department of Biomedical Sciences, Creighton University School of Medicine, Omaha, NE, 68178, United States of America; 3 Department of Otorhinolaryngology—Head and Neck Surgery, University of Nebraska Medical Center, Omaha, NE, 68198, United States of America; 4 Department of Otorhinolaryngology—Head and Neck Surgery, Chinese PLA General Hospital, Beijing, 100853, China; 5 Department of Pathophysiology, Second Military Medical University, Shanghai, 200433, China; 6 Department of Otorhinolaryngology—Head and Neck Surgery, Beijing Friendship Hospital, Beijing, China; Texas A&M University, UNITED STATES

## Abstract

Regulation of gene expression is essential to determining the functional complexity and morphological diversity seen among different cells. Transcriptional regulation is a crucial step in gene expression regulation because the genetic information is directly read from DNA by sequence-specific transcription factors (TFs). Although several mouse TF databases created from genome sequences and transcriptomes are available, a cell type-specific TF database from any normal cell populations is still lacking. We identify cell type-specific TF genes expressed in cochlear inner hair cells (IHCs) and outer hair cells (OHCs) using hair cell-specific transcriptomes from adult mice. IHCs and OHCs are the two types of sensory receptor cells in the mammalian cochlea. We show that 1,563 and 1,616 TF genes are respectively expressed in IHCs and OHCs among 2,230 putative mouse TF genes. While 1,536 are commonly expressed in both populations, 73 genes are differentially expressed (with at least a twofold difference) in IHCs and 13 are differentially expressed in OHCs. Our datasets represent the first cell type-specific TF databases for two populations of sensory receptor cells and are key informational resources for understanding the molecular mechanism underlying the biological properties and phenotypical differences of these cells.

## Introduction

While the genome is identical for nearly every cell in multicellular organisms, the gene expression profile for each cell is different. Diverse patterns of gene expression underlie phenotypic variances of different cell types [1,2]. Transcription factors (TFs) play an essential role in the complex regulation of gene expression patterns in each unique cell type [3]. A TF is a protein that binds to specific DNA sequence motifs, thereby controlling transcription of genetic information from DNA to mRNA [4]. Because cell specificity is enabled by spatial and temporal patterns of gene expression, which in turn are driven by gene regulatory networks [1–6], analysis of TFs in a cell or a cell type is of fundamental importance for understanding the genetic mechanisms that control *in toto* biological properties of the cells under normal and pathological conditions [7]. The critical and unique roles of TFs are highlighted by several studies demonstrating their abilities to reprogram fibroblasts into embryonic stem cells [8,9]. Because of the importance of TFs in genetic regulation, a great amount of effort has been put into the development of resources for the systematic collection, classification and annotation of TFs in genomes from diverse lineages [10–13]. However, a cell type-specific TF database from any normal cell populations is still lacking. A cell type-specific TF database relies on the availability of cell type-specific transcriptomic information. Although a mouse genome-wide transcriptome has been available for more than a decade [14], the availability of cell type-specific transcriptomes is still limited. In the mammalian nervous system, translational profiling is available to only some neurons and sensory receptor cells [15–22].

We recently used DNA microarray technique to analyze transcriptomes of cochlear inner hair cells (IHCs) and outer hair cells (OHCs) isolated from adult mice [22]. IHCs and OHCs are the two types of sensory receptor cells critical for hearing in the mammalian inner ear [23]. Although both types of hair cells (HCs) transduce mechanical stimuli into electrical activity, IHCs and OHCs are morphologically and functionally distinct [23]. The molecular mechanisms that define their morphological and functional specializations are largely unknown. Our transcriptome analyses show that each HC population contains a considerable number of differentially and uniquely expressed genes although a majority of the transcripts are commonly expressed in both HC types [22]. Since TFs control the gene expression profile, it is important to identify what TFs are expressed in IHCs and in OHCs. To date, it is still unclear what TFs are expressed in HCs and what TFs are differentially or uniquely expressed in each population. Although several mouse TF databases based on mouse genome sequences and genome-wide transcriptome are available [11–13], no cell type-specific TF databases have ever been created. We analyzed TF gene expressions in IHCs and OHCs using the HC transcriptome datasets [22]. Since transcriptomes of developing HCs have become available recently [24,25], our analyses also include TF genes from developing HCs. Riken TF Database, TFCat, LocusLink, Gene Ontology Consortium and PubMed were used for verification and reference. Our datasets represent the first cell type-specific TF databases from two specific populations of sensory receptor cells and are key informational resources for understanding the molecular mechanisms underlying these differential properties of HC morphology, function, cell-cycle control and pathology.

## Methods

### 1. Cell collection and transcriptome Analyses

We based our TF analysis on the transcriptome datasets obtained from 2,000 IHCs and 2,000 OHCs collected from adult (P26 to P30) mice. The use and care of animals in this study were approved by Institutional Animal Care and Use Committee of Creighton University. The details of how individual HCs were collected were described in the previous publication [22,26]. A video segment showing how individual cells are collected using the suction pipette technique is attached as S1 Video. As we discussed in the previous publication [22], several steps were taken to avoid contamination by each other and by other cell types in order to obtain highly specific IHCs and OHCs. First, we identified the cells being collected. IHCs and OHCs have unique features and are easily identifiable based on their gross morphology [26] and we collected the cells only when their identity was unambiguous. Second, we collected only solitary HCs that were not attached to any other cell types. Third, we were particularly careful about the suction pressure applied to the pipette to avoid drawing unwanted cells into the pipette. We withdrew the suction pipette (to deposit hair cells) only when the pressure was balanced and no more fluid or cells were being drawn into the pipette. Finally, the chamber, containing the cells, was perfused (with inlet and outlet) with fresh L-15 medium for 5 minutes to wash out debris before the cells were collected using the suction pipette technique. This prevented contamination from lysed cells in the suction pipette. In a separate experiment, we collected isolated OHCs from experimental bath using the suction pipette technique and transferred to a fresh experimental bath (reservoir with continuous perfusion) for additional wash. These cells were then recollected for RNA extraction and microarray analyses. Transcriptome from these cells were presented as OHC4 in the GEO submission. As shown, the gene expression profile was consistent with other OHC groups.

Since three microarray repeats were performed for each cell population, means and standard deviations were calculated. Paired T-tests were done by comparing average intensity values for each transcript from three repeats. p≤0.05 was considered statistically significant when the mean expression value was compared between IHCs and OHCs. As described in the previous study [22], we defined the baseline intensity level at 10.90 for both populations of HCs. A TF gene was regarded as “expressed” if its expression value was ≥ 10.90. We also included analyses of TFs expressed in developing hair cells based on datasets obtained from two recent publications using RNA-seq for transcriptomic analyses [24,25]. The details of how HCs were collected and how RNA-seq data were analyzed are provided elsewhere [24,25].

We constructed mouse HC type-specific TF datasets using annotations from two main mouse TF databases: the Riken TF Database (http://genome.gsc.riken.jp/TFdb) and TFCat (http://www.tfcat.ca). The commercial database TRANSFAC (http://www.gene-regulation.com/ pub/databases.html) [27] currently contains more than 6,692 TF genes and their target genes. Although this database was also used for reference, we prepared our target TF list mainly based on Riken and TFCat databases. This is because the TRANSFAC database has become unwieldy from a genome-wide viewpoint. Most of these resources only collect the TFs and their information in various taxonomies. This practice has led to redundant entry of identical TF genes due to independent deposition from different contributors. Furthermore, alternatively spliced TF genes that are derived from identical genomic loci are also deposited independently. To ensure the comprehensiveness and utility of our reference collection, we compiled a TF list from all TFs listed in the two databases. The union of the two sets yielded 2,230 putative mouse TFs. We then used this list to look for TF gene expressions from the transcriptomes from IHCs and OHCs. Genes that were on this list were selected from transcriptome datasets for further analysis and verification. Entrez Gene, LocusLink, Gene Ontology Consortium and PubMed were used for verification and reference.

### 2. Quantitative RT-PCR

We used qPCR to verify the expression of 20 genes (including those encoding TFs). These genes exhibited differential expression with greater than two-fold difference. For qPCR, additional biological samples of 500 IHCs and 500 OHCs were separately collected from five adult mice (24 to 30 days). Before collecting isolated cells, we first perfused the chamber containing isolated HCs and supporting cells with fresh medium for 10 minutes to wash out debris and RNA from lysed cells in the bath. The cells were collected using micropipette suction technique [26] (in picoliter volume) and re-deposited them in a new chamber with fresh medium. These cells were recollected after their identity was verified under an inverted microscope. By doing this, we further eliminated the possibility of contamination from supporting cells and RNA in the solution. RNA isolation and cDNA transcription was done using the Smart-seq Ultra low Input RNA kit (Clontech Laboratories, Inc., Mountain View, California).

Seven TF genes were chosen to be analyzed with three technical repeats for each gene. Two genes, *Nono* and *Ppia*, were used for reference and normalization. Each gene is equally expressed in both IHCs and OHCs. The oligonucleotide primers were designed using A plasmid Editor (ApE) software (http://biologylabs.utah.edu/jorgensen/wayned/ape/), and utilizing BLAST searches (http://blast.ncbi.nlm.nih.gov/Blast.cgi) to find unique and appropriate sequences with melting temperatures above 60°C that had predicted low rates of homodimerization. The sequences of the oligonucleotide primers are listed as follows: *Bcl2*: Forward primer sequence 5’-3’: ATTGCCAAGAAACGTGTGGCTCC, and reverse primer 5’-3’: GAGCCTCGCTTCACTGCCTCCTTAG; *Clu*: Forward: GCAAGCCCTGCCTGAAGCATAC, and reverse: TGGAAGCTCGGAGGCCCATAGTG; *Fzd4*: Forward: AAATGCTGGGTT GGGAGACGTGTTG, and reverse: AGGTCTCTAGGGTCGGTAAGGTAAG; *Hdac3*: Forward: TCTCACGGCCTGATGATTGTCCCTC, and reverse: TGCAGTTAAGGTTTCAGAGAGC CAC; *Lbh*: Forward: TCTCACGGCCTGATGATTGTCCCTC, and reverse: ACAGAGCAGA GTGGAAGCAAGAG; *Nono*: Forward: GAGAACAAGAGATACGGATGGG, and reverse: TCAATCCAAGGGTTCCATCTG; *Pkrd1*: Forward: CCTCAGTGAGCGTGTCAGCATCC, and reverse: AATGGTGTCCTGGATAGGTCCTG; *Ppia*: Forward: CAAACACAAACG GTTCCCAG, and reverse: TTCACCTTCCCAAAGACCAC; *Six2*: Forward: AGGCAGTTCC GAGGATGAGAAGACG, and reverse: TATCGCCCTCCCACACCGCTTCATC. All primers were acquired from Integrated DNA Technologies (Coralville, Iowa).

Quantitative RT-PCR experiments were run on an Applied Biosystems 7500 Fast Real-Time PCR system. Ten microliters of Powerup SYBR Green Master Mix (Thermo Fisher Scientific, Waltham, MA) was used in each 20 microliter reaction. Primer concentrations were 450 nM. The original cDNA samples were diluted twenty-fold with two microliters for every reaction. The fast thermal cycling mode of the Applied Biosystems 7500 instrument was used, with an initial stage of 2 minutes at 50°C followed by a 2 minute period at 95°C. Sixty-five amplification cycles consisting of 3 seconds at 95°C and 1 minute at 60°C were used. Ct normalization to the two reference genes, *Nono* and *Ppia*, was accomplished by averaging the differences of the two sets of Ct scores between the IHC and OHC samples. The normalized Ct numbers were then inverted (1/Ct) and graphed.

### 3. Immunocytochemistry

Cochleae were perfused with 4% formaldehyde in phosphate buffered saline (PBS) and treated with 0.2% Triton X-100/PBS. Goat serum (10%) was used to block nonspecific binding. The tissue was then incubated with an anti-LBH antibody (Sigma, Lot# HPA034669) and washed with PBS, followed by incubation with secondary antibodies (Life Technologies, Lot# 1579044). A sample was mounted on glass slides with antifade solution (Prolong Antifade Kit, Invitrogen, Carlsbad, CA) before imaging on a Leica Confocal Microscope (Leica TCS SP8 MP). Three cochleae from three adult (P28-P32) mice were used for immunodetection. Two adult cochleae were used as negative control (using only secondary antibody).

## Results

**Fig 1:**
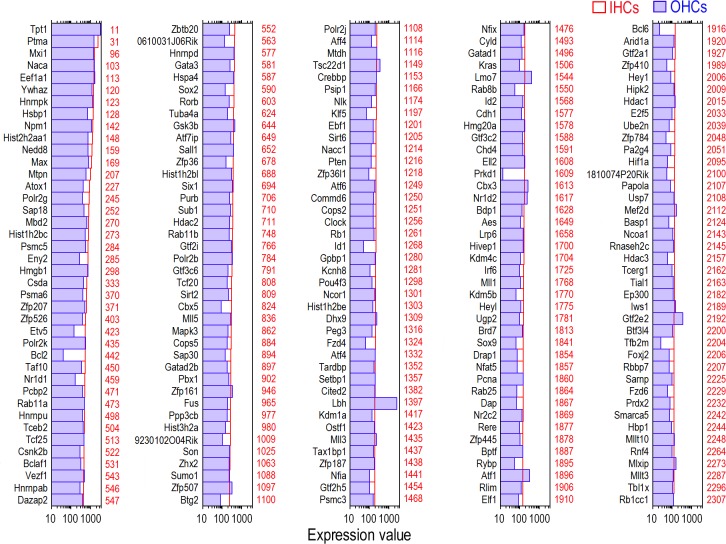
Expression levels of the top 200 TF genes in adult IHCs (in red) in descending order. For comparison, the expression value of the same genes in adult OHCs (in blue) is also presented. Color-coded numbers on the right side of each panel represents the abundance ranking of the each TF among 16,647 transcripts considered to be expressed in adult IHCs.

**Fig 2:**
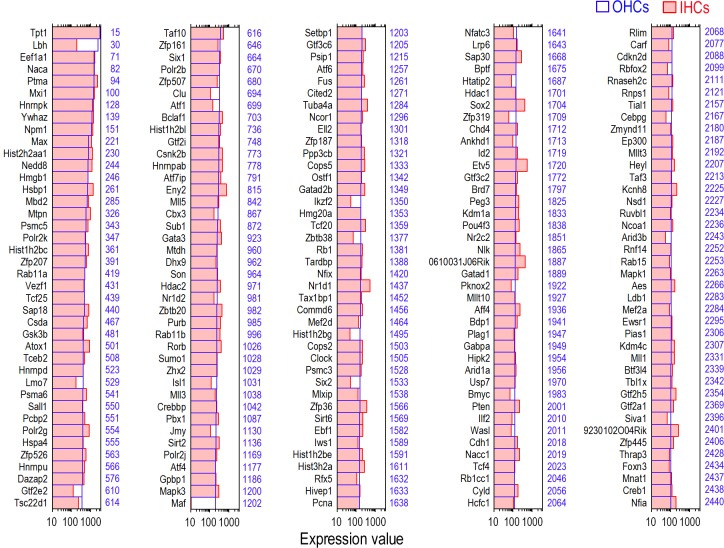
Expression levels of the top 200 TF genes in adult OHCs (in blue) in descending order. For comparison, the expression value of the same genes in adult IHCs (in red) is also presented. Color-coded numbers on the right side of each panel represents the abundance ranking of the each TF among 17,711 transcripts considered to be expressed in adult OHCs.

According to our previous microarray analyses of the gene expression profiles in adult IHCs and OHCs, 16,647 and 17,711 transcripts were expressed in IHCs and OHCs, respectively [22]. To determine what TF genes are respectively expressed in adult IHCs and OHCs, we analyzed the transcripts of each cell population. We included all transcription-related genes—those with DNA-binding properties as well as transcription regulation factors that modulate TFs—as “TFs” in our database. Recent evidence suggests that the contributions of accessory TFs may be equally important in establishing the spatio-temporal regulation of gene activity [28]. Therefore, our databases extend beyond a narrow focus of DNA binding proteins by also including genes involved in histone modifications of the genome. We used the Riken Mouse TF Database and TFCat as the main sources for reference. Among 16,647 transcripts detected in adult IHCs, 1,563 TF genes are expressed. Fig 1 shows the expression levels of the 200 most abundant TF genes in IHCs. Expression levels for the same genes in OHCs are also provided for comparison. To get a better idea of the expression level of the TF genes in comparison to other genes expressed in IHCs, the abundance ranking of the TF genes among the 16,647 genes expressed in IHCs also is presented. There are 17,711 transcripts expressed in adult OHCs. Among them, 1,616 TF genes are expressed. Fig 2 similarly shows the 200 most abundant TF genes in OHCs compared to the same TF genes in IHCs. The abundance rankings of the TF genes among 17,711 genes expressed in OHCs are also illustrated. As shown in Figs 1 and 2, the vast majority of the TF genes richly expressed in one population of HCs are also abundantly expressed in the other. We analyzed TF genes commonly expressed in IHCs and OHCs. Among 16,117 transcripts commonly expressed in both IHCs and OHCs, 1,536 genes were identified as TF genes. The searchable datasets of TF gene expression profiles in adult HCs are presented in S1 Table. Hair cell transcriptomes are available from the National Center for Biotechnology Information-Gene Expression Omnibus (GEO) (GEO submissions number: GSE56866).

**Fig 3:**
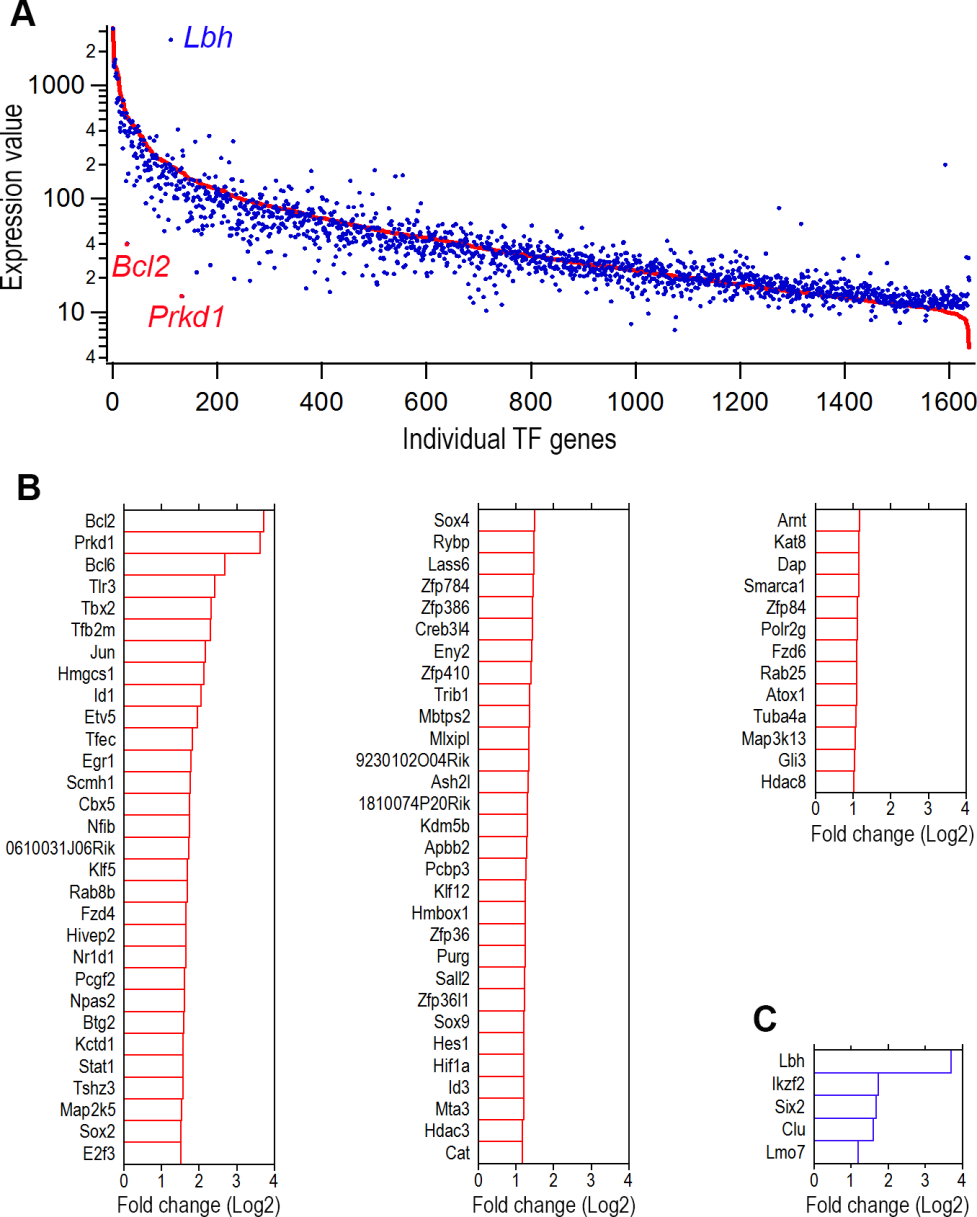
Differentially and uniquely expressed TF genes in adult IHCs and OHCs. (A) All TF genes in IHCs and OHCs. The red line represents the expression level of all individual TF genes of IHCs while each blue dot represents the expression level of the same TF genes in OHCs. TF genes that are differentially expressed in OHCs are above the red line while genes that are differentially expressed in IHCs are below the red line. (B) Differentially expressed IHC TF genes with reference to the expression of same genes in OHCs (in Log_2_). (C) Differentially expressed adult OHC TF genes with reference to the expression of the same genes in IHCs. Only those whose expression levels differ by at least one Log_2_-fold between the two populations of cells are shown in B and C.

We analyzed the TF genes that are differentially expressed in adult IHCs and OHCs, since these TFs are critical for the gene expression profiles that define structures and functions of IHCs and OHCs. We compared the expression levels of all the TF genes in OHCs with those of IHCs. Fig 3A presents an overall picture of TF gene expression profiles in each population. Differentially expressed TFs were categorized as those whose expression levels were above background and at least a twofold different between the two cell types with statistical significance (p≤0.05). There were 73 differentially expressed TF genes in IHCs (Fig 3B). As shown, the top 10 differentially expressed TF genes include *Bcl2*, *Prkd1*, *Bcl6*, *Tlr3*, *Tbx2*, *Tfb2m*, *Jun*, *Hmgcs1*, *Id1*, *and Etv5* with fold difference (in Log_2_) between 1.9 and 3.7. In OHCs there were only five TF genes that met our criteria (Fig 3C). These five TF genes include *Lbh*, *Ikzf2*, *Six2*, *Clu*, *Lmo7* with Log_2_ differences varying from 1.2 to 3.7.

**Fig 4:**
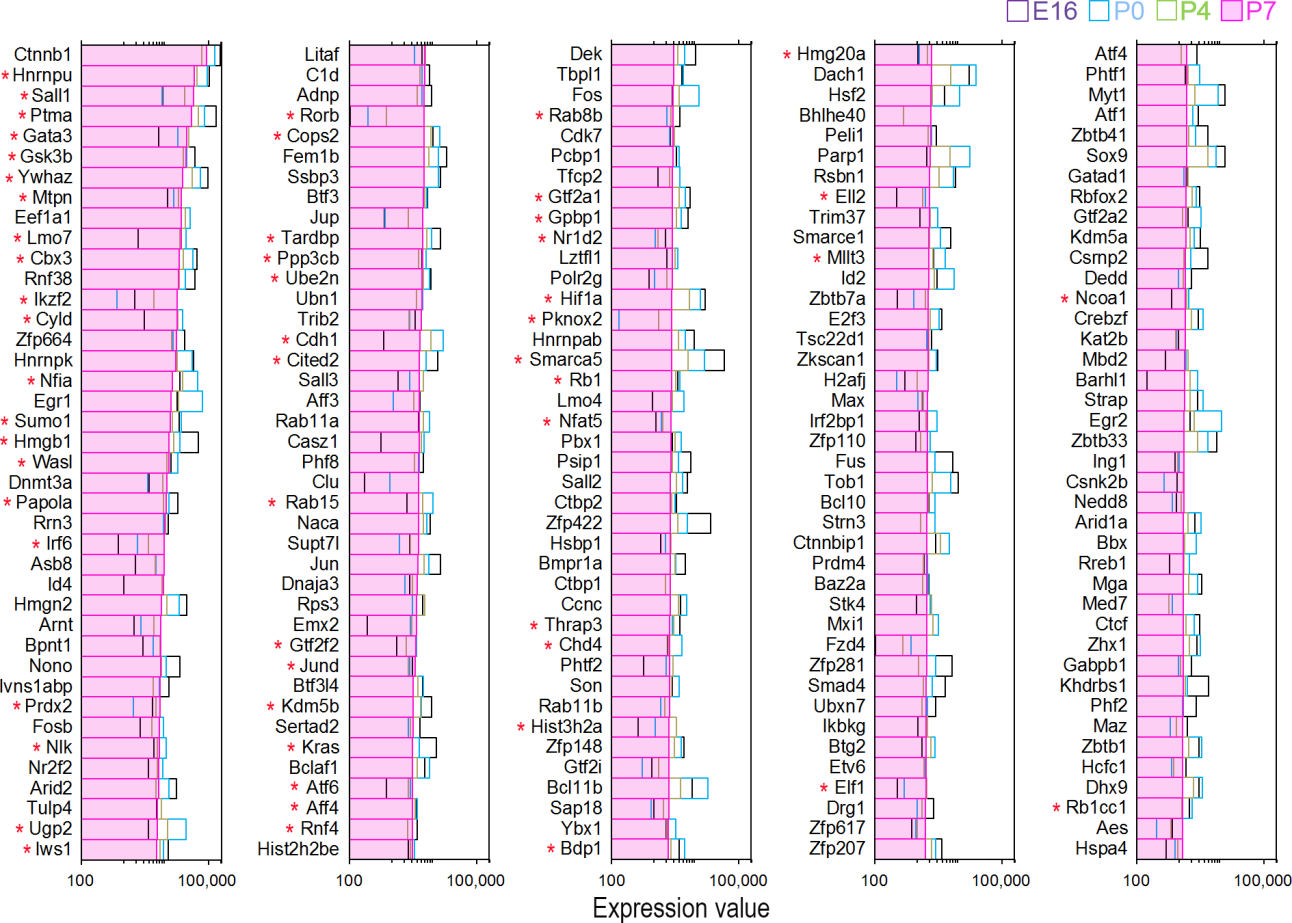
Expression levels of the top 200 TF genes expressed in P7 HCs. The expression of the same TF genes between E16 and P4 is also plotted for comparison. Gene expression profiles at different ages are color-coded. 55 TF genes that are expressed in both P7 and adult HCs are marked by red asterisks.

TFs play critical roles in HC differentiation and maintenance. Two recent studies examined transcriptomes of developing HCs from the mouse cochleae using RNA-seq [24,25]. We analyzed datasets from both studies (GEO accession numbers: GSE65633 and GSE60019) to determine how many TFs are expressed in developing HCs. Using our HC-specific TF dataset as reference, we identified 1,486 TF genes among 19,218 genes expressed in neonatal HCs from the study by Cai *et al*. [24]. From the datasets by Scheffer *et al*. [25], 1,251 TF genes were expressed among 18,199 genes in developing HCs. Since the study by Scheffer *et al*. contains gene expression profiles from four different stages during development, we compared the top 200 enriched TF genes between developing and adult HCs. Fig 4 presents the top 200 TF genes expressed in HCs at postnatal day 7 (P7). The expression of the same TF genes between embryonic day 16 (E16) and P4 is also plotted for comparison. As shown, the TF genes abundantly expressed in developing HCs are quite different from those in adult HCs (Figs 1 and 2 vs. Fig 4). Among the 200 most abundantly expressed TF genes, 55 TF genes are commonly expressed in P7 and adult HCs (marked by red asterisks in Fig 4). The searchable datasets of TF gene expression profiles in developing HCs are also included in S1 Table.

**Fig 5:**
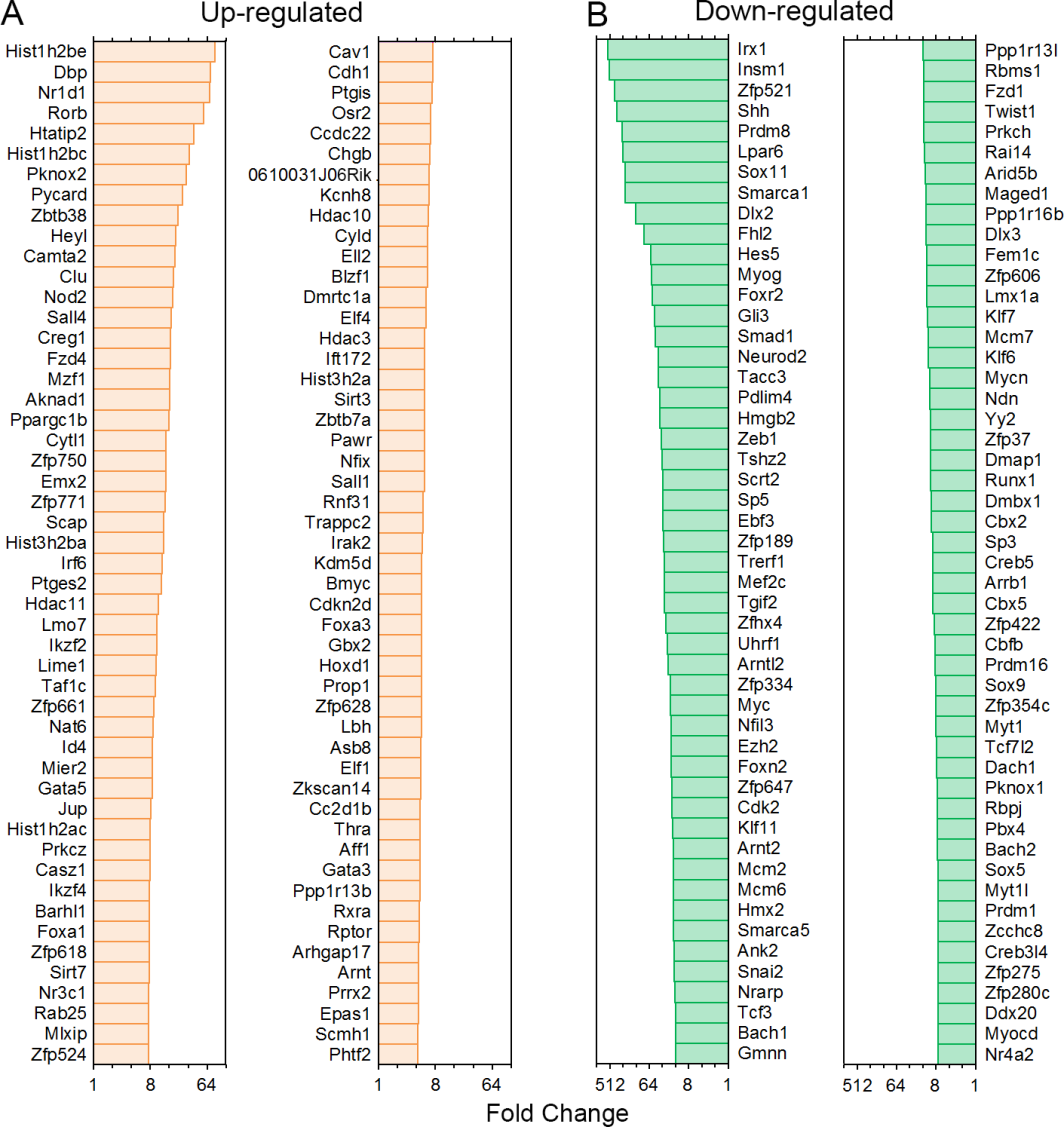
The top 100 TF genes that are up- and down-regulated the expression levels between E16 and P7 were compared with P7 as reference.

**Fig 6:**
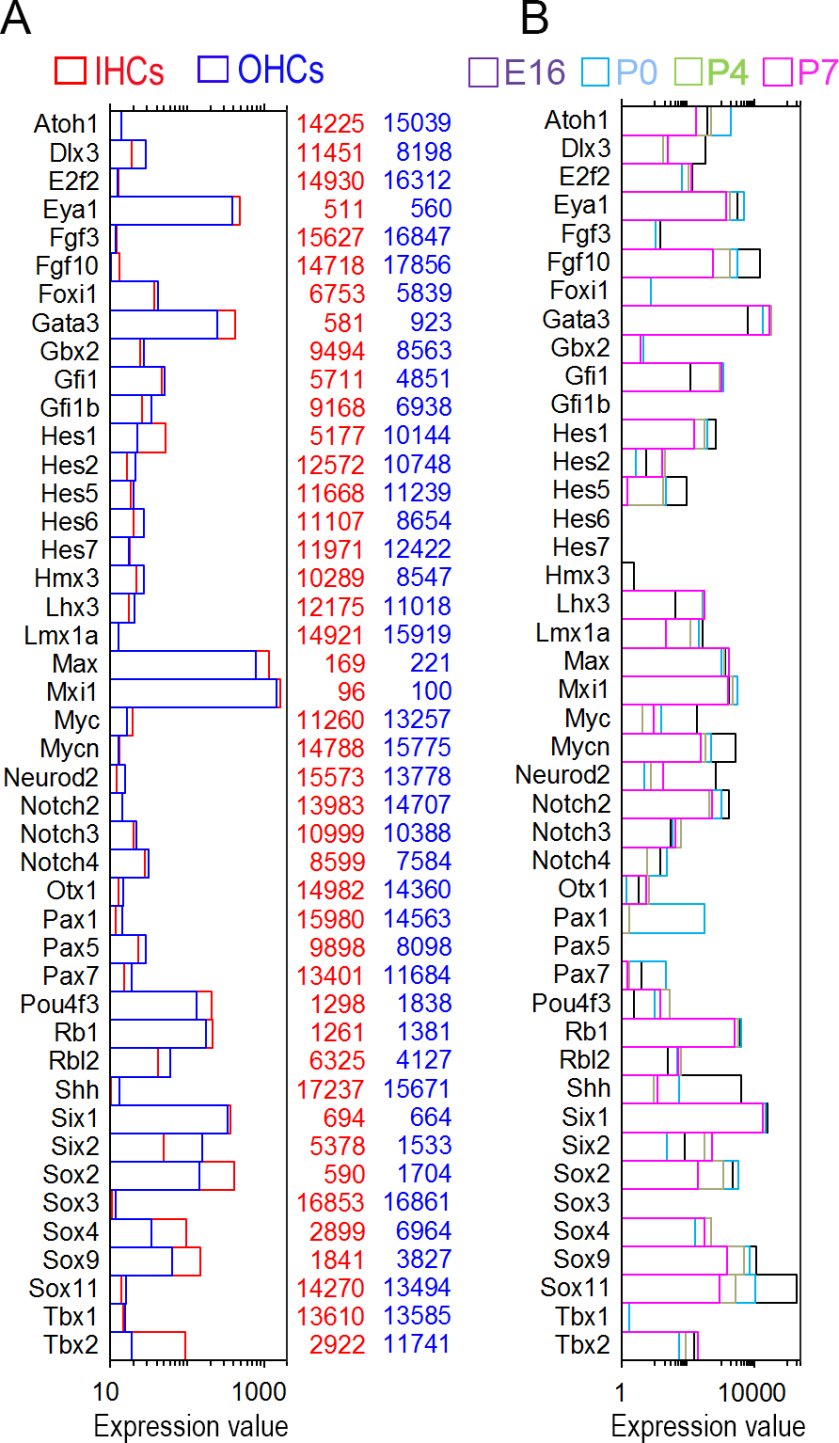
The expression levels of some TFs that are critical for hair cell and/or supporting cell development. (A) TFs in adult IHCs and OHCs. (B) TFs in nascent hair cells.

Using the datasets from Scheffer *et al*. [25], we examined TF genes that are up- and down-regulated during development between E16 and P7. Fig 5 shows the top 100 TF genes that are up- and down-regulated when we compared the expression levels between E16 and P7 using P7 as reference. We also analyzed the expression of TFs that are known to be important for HC differentiation in adult HCs to determine whether they are still expressed. The expression of these TF genes may suggest that they are still required for HC function and maintenance. Fig 6A shows the mean expression levels of those TFs. For comparison, the same TF genes from developing HCs are presented in Fig 6B. As shown, except for a few genes such as *Max*, *Myc*, *Six*, *Pou4f3*, *Rb1* and *Sox2*, most of those TFs are expressed at relatively low levels.

**Fig 7:**
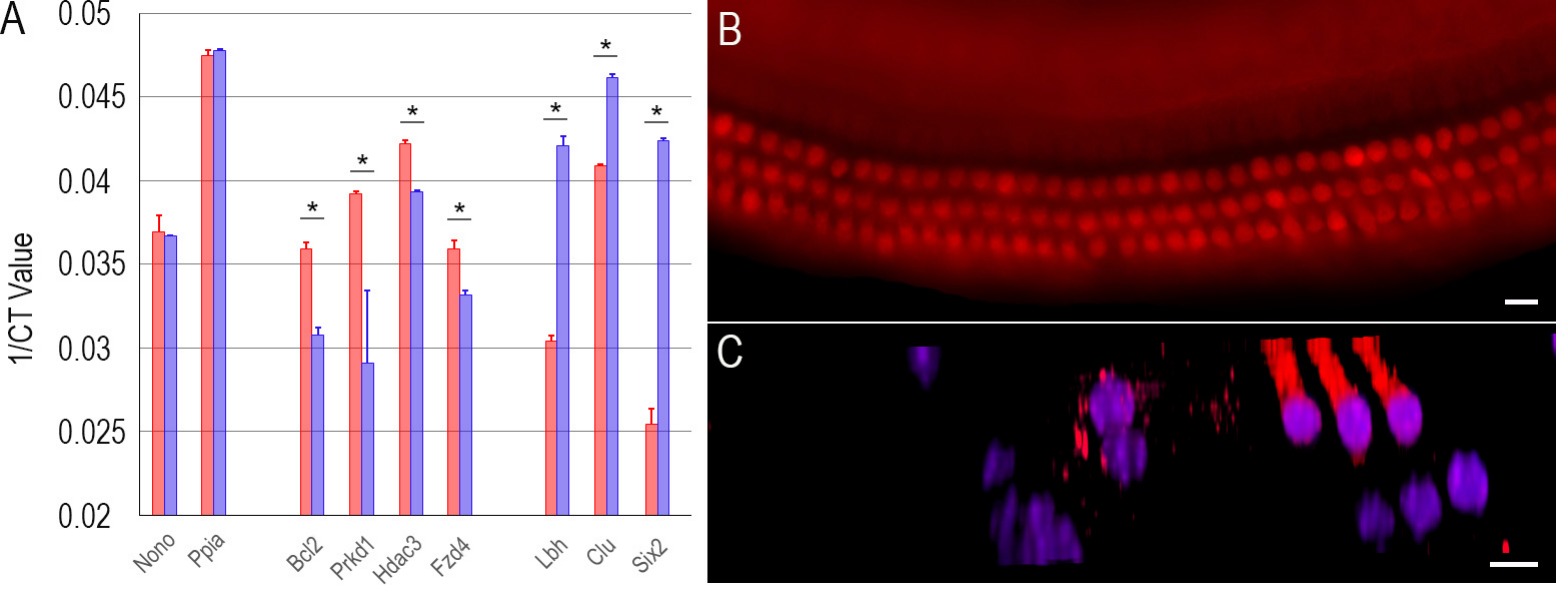
Validation of gene expression by qPCR and immunocytochemistry. (A) Comparison of seven TF genes expressed in adult IHCs and OHCs using qPCR. Note that the ordinate is the inverse of the number of PCR cycles necessary to reach fluorescence threshold. So even a small change (such as 0.01) reflects a large difference. The asterisk represents statistical significance (p < 0.001, Student’s t-test) between each pair. (B) Fluorescence image of expression of LBH in the adult organ of Corti. (C) Optical sectioning of the organ of Corti using confocal microscopy. Nuclei were labelled with DAPI. Bar represents 10 μm.

We used quantitative reverse-transcriptase polymerase chain reaction (qPCR) to verify the expression of 20 genes (including TF genes and other genes such as *Slc26a5*, *Chrna9*, *Chrna10*, *SLC17a8*, *Kcnq4*, *Lmod3*, and *Strip2*). These 20 genes exhibited differential expression with at least a twofold difference according to our microarray datasets [22]. We included other well characterized, differentially expressed genes for qPCR since they can serve as an additional validation. For qPCR, additional biological samples of 500 IHCs and 500 OHCs were separately collected from five adult mice. Our qPCR study showed that these 20 genes were differentially expressed in IHCs and OHCs, consistent with our microarray analyses. Fig 7A shows the expression of seven TF genes in IHCs and OHCs from our qPCR examination. Although the level of expression is normally not comparable between the two techniques (due to different amplification and quantification procedures used in microarray and qPCR), the trend of differential expression of these seven genes is consistent with our microarray data.

LBH is a transcription co-factor that is important for the development of limb bud and heart [29]. Its expression and function in cochlear HCs have never been described before. Our analysis shows that *Lbh* is differentially expressed (~3.7 fold in Log_2_ scale) in OHCs. A significant increase of *Lbh* expression in both cochlear and vestibular HCs was also detected between E18 and P7 [25]. Differential and dynamic expression of *Lbh* may suggest an important role in OHC development and function. We examined the expression of *Lbh* in the adult organ of Corti using antibody-based immunocytochemistry. Fig 7B shows a representative image of LBH expression in the organ of Corti in a whole mount preparation using fluorescence microscopy. As shown, LBH is strongly expressed in OHCs. Optical sectioning (Fig 7C) using confocal microscopy shows that LBH is expressed in the cytosol and nucleus. The expression pattern of LBH suggests that it can be used as a biomarker for HCs.

## Discussion

Gene expression regulation is one of prominent areas in the field of genetics. Regulation of gene expression is essential to determining functional complexity and morphological diversity in different cells and tissues as well as in the wide diversity of species across the tree of life [1–3]. Transcriptional regulation is a crucial step in gene expression regulation and is mediated by TF complexes and availability of genomic DNA. The critical roles of TFs in HC differentiation are highlighted by two studies demonstrating their abilities to reprogram mouse embryonic stem toward HC-like cells [30]. We examined what TF genes are expressed in mouse HCs and describe HC type-specific TF databases. Our datasets differ with other genome-wide TF databases in the following ways: First, our analyses are based on actual gene expression profiles from transcriptome analyses with expression levels and abundance rankings of each TF gene, whereas other mouse TF databases are bioinformatics-based predictions [11,12,31] with no numerical values. Second, our datasets are cell type-specific while datasets are not cell type-specific. In fact, our datasets represent the first TF gene expression database from two specific populations of cells from adult mice. So far, no cell type-specific TF databases of any cell types are available. Third, the two sets of TF gene expression profiles from two purified populations of cells enables us to identify common versus differentially expressed TF genes in these two types of HCs and discern possible gene regulatory networks associated with differential morphological and functional properties.

We show the expression of 1,563 and 1,616 TF genes and their abundance rankings in IHCs and OHCs. These TF genes account for approximately 9% of the total genes expressed in HCs for both populations. The Riken mouse TF database identifies 1,675 TFs [11], among which 939 and 972 are respectively expressed in IHCs and OHCs. The remainder of the TF genes are neither detected nor above the cutoff threshold. This is not surprising since the Riken datasets are based on genome-wide transcriptome of mice while our study identifies TFs only expressed in HCs. We include additional TF genes in our database. This addition reflects the fact that a broader definition of TFs was used in our analyses. Although several mouse TF databases have been created [11,12], there is still no consensus on how many TFs are present in the mouse genome. This reflects the fact that experimental verification of each of the candidate TF genes is still technically impractical. Therefore, up-to-date identification of candidate TFs still largely depends on bioinformatics and predictions based on mouse cDNA sequences. Because a variety of TF identification methods have been used by different investigators, inconsistent criteria and methods used by different studies have resulted in different databases that contain different numbers and annotations of the candidate TFs. For example, the Riken database includes all transcription-related genes, both TFs with DNA-binding properties as well as transcription regulation factors that modulate TFs, for inclusion as TFs [11]. The TFCat database broadly defined a TF as any protein directly involved in the activation or repression of the initiation of synthesis of RNA from a DNA template [12]. Under this definition, 3,230 putative mouse TFs were initially identified. However, further analyses show that fewer than 1,000 TFs were found to have sufficient experimental evidence to be classified either as a TF or as a TF candidate [12]. The initial analyses of the complete human genome sequence [32] estimated that there are between 2,000 and 3,000 TFs. The DBD database includes 1,508 human loci as TFs [33], while it is estimated that there are 1,700 to 1,900 TF-coding genes in the human genome with a high-confidence data set of 1,391 genomic loci [7]. Another study using an ORFeome-based analysis of human TF genes estimated that there are 1,962 TFs [34]. We would like to add that it is likely that the actual number of TF genes in HCs may be greater than what we currently report here. This is because approximately a proportion of transcripts identified in HC transcriptomes have not been characterized. It is very likely that some of those transcripts are TF genes.

An advantage of our study is the ability to compare the expression of TF genes between the two types of HCs, which share many common features and yet are morphologically and functionally different. This enables us to identify common versus unique TFs in these HC types. While identification of the common genes is important to ascertain TFs that regulate gene expression for shared structures and functions (such as stereocilia bundles, mechanotransduction, and synaptic machinery), identification of differentially expressed TFs reveals gene regulation for the unique morphological and functional properties of IHCs and OHCs. Although the roles of the majority of these commonly and differentially expressed TFs in HCs are not yet characterized, identification of common and differentially expressed TFs is the first step that can lead to characterization of the TFs that are important to HC functions. For example, recent evidence suggests that TMC1 may be a component of the mechanotransduction channels [35,36]. What TF regulates the *Tmc1* gene expression is unknown. Since both types of HCs are mechanosensitive, the TF that regulates *Tmc1* gene must be present in both IHCs and OHCs. Conversely, *Slc26a5*, the gene that encodes motor protein prestin, is significantly differentially expressed in OHCs [22,37]. The TF regulating *Slc26a5* expression is expected to be differentially expressed in OHCs.

Although the specific function of each of the TFs in HCs is not known, some possible function may be inferred from their known function revealed in other cells. Three TFs that are differentially expressed in IHCs may offer some clue for understanding why there is a differential vulnerability to ototoxic drugs and noise insult between IHCs and OHCs [38,39]. Noise trauma, aging, and ototoxicity preferentially damage the OHCs, leading to increased hearing thresholds and poorer frequency resolution [39,40]. BCL6, an evolutionarily conserved zinc finger TF, is generally regarded as an important anti-apoptotic regulator [41]. As shown in Fig 3B, *Bcl6* expression in IHCs is 2.7-fold greater than in OHCs. BCL2 also regulates cell apoptosis and is specifically considered as an important anti-apoptotic protein [42]. Some evidence suggests that BCL2 is also a transcription suppressor [43]. The expression of *Bcl2* in IHCs is 3.7 Log_2_ greater than in OHCs. Clusterin, encoded by *Clu*, is a 75–80 kDa disulfide-linked heterodimeric protein associated with the clearance of cellular debris and apoptosis. One isoform of CLU, localized in the nucleus, can induce apoptosis [44]. As shown in Fig 3C, *Clu* expression in OHCs is 1.6 Log_2_ greater than in IHCs. Differential expression of anti-apoptotic *Bcl6* and *Bcl2* in IHCs and differential expression of pro-apoptotic *Clu* in OHCs may underlie the molecular mechanism that predisposes OHCs to being more susceptible to apoptosis and aging than IHCs.

Previous studies have identified a number of TFs that are critical for HC differentiation and development [45]. We show that a majority of those TF genes are still expressed in adult HCs although at relatively low levels (Fig 6A). The fact that those TF genes are still expressed in adult HCs suggests that they may play an important role in HC function and maintenance. For example, our datasets show that *Atoh1* is still expressed in adult IHCs and OHCs. Atoh1 is a TF that plays a critical role in HC differentiation [46]. *Atoh1* is highly expressed in HCs during inner ear development. Embryonic *Atoh1*-null mice fail to generate cochlear and vestibular HCs [46]. Terminating *Atoh1* expression after birth results in a complete loss of HCs in less than four weeks [47]. Low level expression of *Atoh1* shown in our study supports the notion that *Atoh1* expression in adult HCs is required for HC survival [47,48]. Two members of the *SoxC* family, *Sox4* and *Sox11*, are downregulated during development (Figs 5 and 6). A recent study shows that conditional deletion of *Sox4* and *Sox11* results in loss of HCs and abnormal development of the organ of Corti. Increased expression of these two TFs restores supporting cell proliferation and the production of new HCs in adult vestibular epithelia [49]. *Sox2* is also required for the development of inner ear sensory domains [50]. Mutants for *Sox2* have absent or disordered formation of sensory domains and HCs [50]. *Sox2* is detected in both HCs and supporting cells before birth [51]. In the adult organ of Corti, SOX2 is detected only in the supporting cells using immunocytochemistry [51]. This is in contrast to our microarray analyses, which show that *Sox2* is still expressed in adult (P26 to P30) HCs. The discrepancy is actually not surprising since the levels of gene expression and protein expression may be attributed to the role of miRNAs, which function in RNA silencing and post-transcriptional regulation of gene expression [52]. *Sox2* is a predicted target of miR-183 family [53], which is abundantly expressed in HCs, especially in IHCs [53]. The relative higher level of expression of *Sox2* in IHCs is consistent with relative higher level expression of miR-183 in IHCs [53]. Besides, immunocytochemistry is not as sensitive as PCR-based techniques. Proteins that are expressed at low levels are often missed by antibody-based immunocytochemistry. A recent study using RNA-seq also detected relatively high-level expression of *Sox2* in vestibular HCs at P16 [25]. Finally, our recent unpublished study using RNA-seq to characterize transcriptomes of pillar cells, Deiters’ cells, IHCs, and OHCs from three-month-old mice confirms that *Sox2* is still expressed in IHCs and OHCs. Thus, it is possible that, like *Atoh1*, a low level expression of *Sox2* may be necessary for HC survival and function in adult HCs.

The expression of a number of TFs may offer some important clues to understand why adult mammalian HCs have lost the capacity to proliferate. The proliferation and development of embryonic HCs are impacted by the presence of N-Myc paralogs [54]. MYC belongs to an extended TF network involving *Myc* paralogs and MYC-associated protein genes, referred to as the *Max* and *Mlx* genes [55]. In adult cochlear HCs very low levels of *Myc* and *Mycn* transcripts are present, while *Max*, the obligate binding partner of the MYC proteins, is expressed at ~60 fold greater levels. A heterodimer between MAX and the MYC paralogs are necessary to bind E-box elements for gene transcription [56]. MAX homodimers and MAX heterodimers with the *Mxd* and *Mnt* proteins will lead to cycle-cycle arrest and may also contribute to differentiation [56]. Three of the four *Mxd* paralogs, *Mxd1*, *Mxd3*, and *Mxd4*, as well as *Mnt* are expressed at low levels; whereas *Mxi1* (alias–*Mxd2*) is ~1.5–2 Log_2_ higher than *Max* in IHC and OHC expression. The presence of MAX and MXI1 in HCs should provide significant cell-cycle arrest and are likely impediments to the successful realization of therapeutic approaches using cell-cycle regulatory genes, including *Myc* itself [57].

Finally, the TF databases of the two types of HCs presented here will likely provide many opportunities for a broad range of further research into transcriptional regulation of gene expression in HCs. The longitudinal and radial gradients of gene expression in cochlear HCs should be particularly interesting since it may contribute to the understanding of the quantitative-based regulatory mechanisms of TFs and miRNAs. So far we know very little about the biological processes that most of these TFs mediate. By indicating which TFs are present in IHCs and in OHCs we provide a starting point for future studies into the activity of individual TFs and how groups of TFs mediate HC phenotype and regulate differentiation, cell cycle control and survival. Understanding HC fate determination, cell cycle regulation and long-term maintenance are essential for developing strategies for HC repair, regeneration and maintenance, all of which are critical for maintenance of HCs as well as restoring lost hearing using gene therapy [58,59].

## Supporting Information

S1 Video Video showing an OHC isolated from the apical turn of an adult mouse being drawn into the suction pipette. (MOV)

S1 Table A complete set of transcription factors expressed in IHCs and OHCs. (XLS)
